# Grit in the Workplace Experienced by Taiwanese Adults With Congenital Heart Disease: A Phenomenological Study

**DOI:** 10.1111/jocn.70051

**Published:** 2025-07-22

**Authors:** Yu‐Shiu Liu, Chun‐Wei Lu, Hung‐Tao Chung, Jou‐Kou Wang, Ying‐Mei Shu, Chi‐Wen Chen

**Affiliations:** ^1^ Department of Nursing MacKay Medical University New Taipei City Taiwan; ^2^ Department of Pediatric Cardiology National Taiwan University, Children's Hospital Taipei Taiwan; ^3^ Department of Pediatrics Chang Gung Children's Hospital Taoyuan Taiwan; ^4^ Department of Nursing Chang Gung University of Science and, Technology Taoyuan Taiwan; ^5^ College of Nursing National Yang Ming Chiao Tung University Taipei Taiwan

**Keywords:** adults with congenital heart disease, grit, nursing, phenomenology, work

## Abstract

**Aim:**

To explore how adults with congenital heart disease (ACHD) experience and express grit in the workplace.

**Design:**

Qualitative study using Husserl's descriptive phenomenology.

**Methods:**

Between March 2022 and June 2023, semi‐structured interviews were administered to 18 ACHD recruited from two medical centre outpatient departments. The collected data underwent analysis utilising Colaizzi's 7‐step analysis method, coupled with Lincoln and Guba's framework, to ensure credibility and trustworthiness.

**Results:**

The analysis revealed five prominent themes derived from the data: (a) career choices amid constraints; (b) adjustments to one's work environment for reasons of fatigue; (c) crises in the workplace arising from exceeding one's physical limits; (d) supportive networks for better health and job stability; (e) resilience at work for balance and fulfilment in life.

**Conclusion:**

Grit significantly influences life satisfaction and job performance among adults with congenital heart disease, highlighting its profound impact on their experiences. Patients exhibit perseverance in job pursuits, adapt work methods to manage physical fatigue, confront challenges during work crises, value family and societal support and aim for self‐satisfaction. These findings highlight the impact of grit and mental health on ACHD's lives and work, providing insights for better psychological support and interventions.

**Implications for the Profession:**

This study clarifies the need for healthcare professionals to incorporate workplace grit training and assessment into ACHD care.

**Impact:**

Recognising grit as a key factor in ACHD patients' lives informs holistic care, workplace inclusivity and policies that enhance their long‐term well‐being.

**Reporting Method:**

This study was performed in accordance with the COREQ guidelines.

**Patient or Public Contribution:**

No patient or public involvement.

**Summary**

- What does this paper contribute to the wider global clinical community?
  ○ The study identified five themes of grit experiences in the workplace for ACHD.
  ○ This research provides experiential data on how grit enhances job and life satisfaction for ACHD.
  ○ Grit plays a crucial role in providing psychological support and intervention for ACHD in both life and work.

## 1 Introduction

The number of adults with congenital heart disease (ACHD) is increasing because of improved survival rates among individuals with complex conditions (Bonanni et al. 2025). As congenital heart disease (CHD) becomes a chronic condition, many ACHD enter the workforce; however, their careers and everyday life are substantially affected by ongoing challenges in their health. Grit, a psychological trait defined by perseverance and passion for long‐term goals (Duckworth et al. 2007), is a key predictor of success in various aspects of life, especially in the workplace. Grit is positively associated with life satisfaction, well‐being, achievement and learning (Sigmundsson and Haga 2024). Previous studies on ACHD have focused on health behaviours (Holbein et al. 2020; Ko et al. 2019), resilience (Steiner et al. 2023), and individual or social resources (Hardy et al. 2021; Khan et al. 2021). However, when life and work intertwine, the significance of long‐term goals and perseverance in achieving life and job satisfaction becomes more evident. While quantitative research has identified grit as a mediator promoting healthy lifestyles and reducing depression in ACHD (Liu, Chung, et al. 2024; Liu, Lu, et al. 2024), it fails to capture how grit is perceived, experienced, and given meaning over time in daily life and the workplace. Using a phenomenological qualitative approach, this study explores the essence of ACHD's lived experiences, examining how they redefine ‘success’ and ‘grit’ to cope with illness‐related challenges, adjust personal and career goals, and express grit in diverse ways. This research provides critical insights into the psychosocial and healthcare needs of ACHD, informing future strategies to enhance their quality of life.

## 2 Background

Advances in early surgical interventions and health care have improved the survival rate of newborns with CHD, enabling many to reach adulthood. However, because of the difference between chronological and physiological age, ACHD have increased risks of early‐onset complications and frailty syndromes. Chronic inflammatory responses, driven by lifelong stressors such as haemodynamic abnormalities and repeated surgeries, contribute to premature ageing in this population (Bonanni et al. 2025). Although many ACHD achieve clinical stability, they remain susceptible to both cardiac and non‐cardiac complications, particularly heart failure, which is more prevalent in younger patients with CHD than in older populations (Kheiwa et al. 2025). Atrial arrhythmias are among the most common complications in ACHD, and the incidence of atrial fibrillation is increasing, especially among those with heart failure. The underlying pathophysiology likely involves congenital defects, surgical patches and haemodynamic abnormalities (Kerley et al. 2025). Atrial arrhythmias often precede the onset of heart failure by several years. Although radiofrequency ablation is considered an effective treatment, its success rate is low and the recurrence rate is high in the presence of heart failure (Lauwers et al. 2025).

Although survival rates among ACHD have improved, these individuals continue to experience higher unemployment rates and face substantial challenges in securing and maintaining employment. Participation in the workforce plays a critical role in fostering life satisfaction, a sense of meaning and emotional well‐being (Hoyos et al. 2024). Although most ACHD are capable of entering the workforce, these patients are more likely to remain unemployed than their counterparts in the general population, with unemployed individuals reporting and those without employment having a lower quality of life (Enomoto et al. 2020). Studies have reported no significant differences in educational attainment or employment rates between young patients with CHD and their peers in the general population. However, ACHD have lower overall employment rates and are more likely to occupy lower‐level positions compared with their peers in the general population (Pelosi et al. 2023). Living with chronic illnesses, such as CHD, affects one's professional life, often necessitating a supportive workplace culture that encourages open communication and reasonable accommodations (Bosma et al. 2021). Thus, interventions that are tailored to ACHD must be grounded in an understanding of their workplace experiences.

Adults with CHD live with a lifelong chronic condition that poses ongoing challenges to their quality of life. Although medical advancements have extended life expectancy, the prognosis of CHD remains uncertain, particularly among younger individuals. Frequent hospitalisations and medical interventions contribute to considerable stress (Freiberger et al. 2023), indicating the importance of addressing psychosocial well‐being. Previous studies have examined resilience in adults with CHD, focusing on personal resources such as optimism, self‐initiated activities, social support and a sense of purpose (Steiner et al. 2023). However, resilience under conditions of high stress remains insufficiently understood, highlighting the need for further investigation to inform the development of effective psychological support strategies.

Grit, defined by perseverance and a passion for a set of long‐term goals (Duckworth et al. 2007), is a predictor of success in multiple domains, including education, employment and well‐being. Studies have reported an association of grit with improved quality of life, decreased stress (Knauft et al. 2024), and enhanced self‐management (McKelvey et al. 2023), especially among individuals with chronic illnesses. The positive effects of grit have been observed in populations such as young patients with cancer (McKelvey et al. 2023), university students with type 1 diabetes (Tsevat et al. 2025), and individuals with multiple sclerosis (Lee et al. 2022), where it supports emotional regulation, resilience and pain tolerance (Buckingham and Richardson 2021). While existing studies highlight the positive effects of grit in managing emotional challenges and health‐related adversity, less is known about how grit manifests in the workplace among individuals living with chronic illness, particularly ACHD. Unlike educational or clinical settings, the workplace introduces unique stressors such as performance expectations, career advancement pressures, job security and workplace stigma related to health conditions (McGonagle et al. 2024). Understanding the experiences of individuals with chronic illnesses in the workplace is an important area that healthcare providers need to address (Farre et al. 2023). Thus, exploring grit through the lens of workplace experience is critical for understanding how individuals maintain a sense of purpose, agency and identity in the context of chronic illness.

Therefore, this study aimed to explore the following research questions: How did ACHD experience and express grit in the workplace context, and how did they redefine success and perseverance in response to the challenges of living with a chronic condition?

## 3 The Study

This study aimed to explore how ACHD experience and express grit in the workplace.

## 4 Methods

### 4.1 Design

This study employed a Husserlian approach to descriptive phenomenology (Husserl 2001).

### 4.2 Theoretical Framework

This study is grounded in Husserlian descriptive phenomenology, which seeks to uncover the essence of lived experiences by exploring how individuals perceive, interpret and give meaning to their world (Husserl 2001). Although grit is traditionally conceptualised as a psychological trait—reflecting perseverance and passion toward long‐term goals—it is increasingly recognised as context‐sensitive, shaped by personal history, cultural norms, social roles and environmental demands (Klappa et al. 2022, 2020). In the case of ACHD, grit is not only a measurable characteristic, but a lived phenomenon expressed through occupational choices, adaptive behaviours and identity negotiations in the face of chronic illness.

Phenomenology offers a valuable lens for examining grit beyond trait‐level definitions, allowing us to explore how grit is actually experienced, constructed and sustained within the lived realities of participants. The core concepts of intentionality, essence and bracketing guided this inquiry (Watson 2024). This study used intentionality to explore how individuals with ACHD direct effort and meaning toward work despite fluctuating health. The search for essence allowed us to distil the lived meaning of grit, rather than relying on assumed definitions, while bracketing helped minimise researcher bias and preserve the authenticity of participants' voices. Through this process, we systematically identified shared themes that reveal how grit manifests in the real‐world work‐life experiences of individuals with ACHD. These findings offer valuable insights for nursing care by highlighting lived strategies of persistence and adaptation that may be overlooked in traditional trait‐based assessments.

### 4.3 Study Setting and Recruitment

This study employed purposive sampling to recruit employed ACHD from paediatric and adult CHD outpatient clinics at two medical centres in northern Taiwan. Purposive sampling enabled the targeted selection of individuals who had firsthand experience of the phenomenon under investigation—grit in the workplace—and could provide rich, in‐depth descriptions relevant to the research aim. Recruitment continued until data saturation was reached, which was operationally defined as the point at which no new themes, subthemes, or meaningful variations emerged during three consecutive interviews (Palinkas et al. 2015). This decision was confirmed through ongoing iterative analysis and team discussions. Prospective participants were included if they had (1) a confirmed diagnosis of CHD before the age of 2 years, (2) were aged between 20 and 59 years, (3) were employed full time, defined by more than 35 h of work per week (Geyer et al. 2009), (4) were literate and able to communicate in either Mandarin Chinese or Taiwanese dialect and (5) voluntarily provided their written informed consent. Pregnant women and individuals with intellectual disabilities or chromosomal abnormalities were excluded from the study.

### 4.4 Ethical Considerations

This study was approved by the Medical Ethics Committees of Chang Gung Memorial Hospital on 26 January 2022 (No: 202101631B0C601) and National Taiwan University Hospital on 24 February 2022 (No: 202012276RINB). Participants received both written and verbal explanations of the study's purpose and were informed that their participation was entirely voluntary, ensuring adherence to ethical standards. Participants were assured that they could withdraw their consent or leave the study at any time with no repercussions. Anonymity was strictly maintained, and all data were used solely for this study. To protect the participants' privacy, personal identifiers were removed or modified as extensively as possible without compromising the integrity of the content. Interviews were recorded only with the participants' explicit consent. Given the sensitive nature of the topics discussed, every effort was made to minimise psychological distress. In addition, all names and identifying information were excluded from the data, and each participant was assigned a unique identification code. The list linking participant IDs to personal data was stored separately to ensure confidentiality.

### 4.5 Data Collection

Between March 2022 and June 2023, each participant participated in one unstructured interview lasting approximately 30–60 min. Eligible participants were informed of the study's purpose, significance and voluntary nature. Before the interview proper, data on background characteristics were collected, namely, age, gender, marital status, educational level, employment status, disease course and self‐reported health status. This information was essential for analysing the relationship between these background characteristics and the role of grit in the workplace in particular and life in general. Due to COVID‐19 restrictions during the study period, two participants were interviewed in person, while the remaining 16 were interviewed via synchronous online video conferencing (e.g., Zoom or Microsoft Teams). To ensure consistency and control for potential differences in data quality, all interviews were conducted by the same trained interviewer using a standardised semi‐structured interview guide (Table 1). Participants were encouraged to select a quiet, private location for the interview, and audio‐visual quality was checked prior to each session. Field notes were taken during and after all interviews to capture non‐verbal cues and contextual observations, regardless of modality. The interviewer's reflexivity and adherence to methodological rigour helped ensure comparability in the depth and richness of the data collected across both modalities. All interviews were conducted in participants' native language, and audio data were transcribed verbatim and analysed within 24 h. During the interviews, the first author documented nonverbal cues, emotional expressions and personal reflections. When analysing the transcripts, the first author followed the participants' stream of consciousness to explore the essence of grit in the work‐life experiences of ACHD. Data from the fifteenth participant indicated that saturation had been reached. However, because of a lower number of male participants among the first 15 interviews and to enrich the dataset, the final three participants were all men.

**TABLE 1:** Interview guide.

Questions	
1.	Please introduce yourself briefly, including your understanding of your congenital heart disease and your current health condition.
2.	Since turning 20, have you experienced any major life events? Could you share your experience of these events? Do you consider them setbacks, or have you encountered other challenges in life? How did you navigate these experiences?
3.	Could you share your current life goals from the age of 20 until now? What life experiences ignite your passion? What experiences have helped you persevere? Anything else?
4.	Could you describe your work experiences from age 20 until now?
5.	What motivated you to choose your current profession? What are your aspirations or vision for this career? Have any internal thoughts or external factors ever caused you to reconsider your initial motivation?
6.	Before entering your current profession, did you try other jobs? What was the experience like? Did you have any visions for your career, and how did you work toward maintaining those goals?
7.	In your everyday life, how do you express passion for your current profession? What experiences help you stay committed to your work? Anything else?
8.	Have you developed other personal interests or passions? Have you ever considered giving up on your career aspirations? How have you planned and taken steps to pursue your career goals?
9.	Finally, what advice would you give to other adults with congenital heart disease regarding their careers or work?

### 4.6 Data Analysis

The study data were analysed using Colaizzi's seven‐step method (Colaizzi 1978), which is based on Husserlian phenomenology. In Step 1, the audio recordings were transcribed verbatim and read multiple times to ensure that the researcher was familiar with the content. In Step 2, a comprehensive understanding of grit in the work and life experiences of ACHD was developed, and key descriptions illustrating how grit contributed to meeting work‐related demands were extracted from the transcripts. In Step 3, the extracted content was interpreted, and any ambiguities were clarified through follow‐up discussions with participants, enabling the addition of detailed explanations. In Step 4, significant statements were formulated into meaningful categories, which were subsequently organised into subthemes and overarching themes. The validity of these themes was reviewed repeatedly through cross‐checking with the original transcripts. In Step 5, the themes were closely linked to the phenomenon under investigation, and exhaustive descriptions were developed. In Step 6, the transcripts were re‐examined to confirm that the structured themes and subthemes accurately reflected the essence of grit in the work and life experiences of ACHD. In Step 7, the final results were presented to all participants for validation; three participants confirmed that the thematic structure accurately represented their experiences, thereby supporting the credibility of the findings. Step 1 was performed by the first author, whereas Steps 2 through 7 were performed in collaboration with the corresponding author until consensus was reached between both authors. After Step 3, the fifth author, an external expert, joined the review process to conduct an expert audit, and investigator triangulation was applied. Each author independently coded transcripts and then met to compare interpretations. Discrepancies in coding or theme development were discussed in analytical meetings and resolved through consensus, referring back to the data when needed. Memos and reflexive notes were used to ensure transparency and minimise personal bias, thereby enhancing objectivity and trustworthiness. To maintain bracketing, the researcher consistently described how they engaged with the data throughout the analytical process.

### 4.7 Rigour and Reflexivity

This study maintained rigour by employing the criteria of credibility, transferability, dependability, and confirmability in accordance with Lincoln and Guba's framework (Denzin and Lincoln 2011). To enhance credibility, all interviews were conducted by the same researcher using a consistent semi‐structured interview guide. The interviewer engaged in prolonged engagement with participants, fostering trust and enabling the collection of authentic, in‐depth data. Data saturation was ensured through iterative data collection and analysis. Transferability was supported by detailed descriptions of the research context and participant characteristics, and by validating the findings with three participants. Their feedback confirmed that the interpretations accurately reflected their lived experiences. Dependability was established through an audit trail and peer review of the coding and thematic development. Coding decisions were discussed among the research team to ensure consistency and analytic rigour. Confirmability was strengthened through investigator triangulation, reflexive journaling and external expert audit. An independent qualitative research expert reviewed selected transcripts, codes and thematic structures, offering feedback that helped refine the final analysis. Reflexivity was maintained throughout. The first author, who had prior clinical experience with ACHD patients, acknowledged her preunderstandings regarding patients' resilience and work challenges. These assumptions were bracketed through reflective memo writing and regular discussions with co‐authors to ensure that emerging interpretations remained grounded in participant narratives rather than the researcher's clinical background. The Consolidated Criteria for Reporting Qualitative Research (COREQ) checklist (Tong et al. 2007) was used to ensure transparent and comprehensive reporting of the study data (Supporting Information S1).

## 5 Findings

### 5.1 Characteristics of Participants

A total of 18 eligible ACHD (9 men and 9 women) participated in this study. The participants' age ranged from 23 to 43 years, with a mean age of 34 years. In terms of educational background, 94.44% had attained a university‐level education or higher. In addition, 50.00% of the participants were married. Their years of experience in the workforce at the time of the study ranged from 3 months to 21 years. Three participants (16.67%) were employed in the manufacturing industry, three (16.67%) held general administrative roles, and three (16.67%) worked in the hospitality industry. The most common diagnosis among participants was tetralogy of Fallot, which affected eight (44.44%) individuals. Moreover, 66.67% of the participants had a New York Heart Association (NYHA) classification of class II and III (Table 2).

**TABLE 2:** Sociodemographic characteristics of the participants (*n* = 18).

Variable	Mean ± SD/*n* (%)
Age (year)	34.28 ± 4.55
20–30	4 (22.22)
31–40	12 (66.67)
41–50	2 (11.11)
Gender	
Man	9 (50.00)
Woman	9 (50.00)
Educational level	
Senior high or less	1 (5.56)
College or higher	17 (94.44)
Marital status	
Unmarried	9 (50.00)
Married	9 (50.00)
Employment^a^	
Manufacturing	3 (16.67)
General occupations	3 (16.67)
Hospitality industry	3 (16.67)
Public utilities	2 (11.11)
Information technology industry	2 (11.11)
Service industry	1 (5.56)
General business	1 (5.56)
Healthcare	1 (5.56)
Educational and cultural institutions	1 (5.56)
Transportation industry	1 (5.56)
Diagnosis	
Tetralogy of fallot	8 (44.44)
Atrial/ventricular septal defect	3 (16.67)
Transposition of the great arteries	3 (16.67)
Single ventricle defects	1 (5.56)
Double outlet right ventricle	1 (5.56)
Congenital anomaly of the coronary artery	1 (5.56)
Ebstein's anomaly	1 (5.56)
NYHA class	
I	6 (33.33)
II	11 (61.11)
III	1 (5.56)

Abbreviations: NYHA, New York Heart Association; SD, standard deviation.

^a^ Based on the standard industrial classification system of the Republic of China.

### 5.2 Main Findings

An analysis of the interpreted narrative data yielded a total of 554 meaningful units, which were organised into 26 categories. These categories were further classified into 17 subthemes and subsequently grouped into five overarching themes. These themes represented the lived experiences of grit in the workplace among ACHD (Table 3). The following section presents each main theme and its associated subtheme clusters along with the corresponding categories. Within each subtheme cluster, the categories are illustrated using quotes (given with the respective participant code and meaning unit).

**TABLE 3:** Themes, subthemes and representative quotes.

Theme	Subtheme/quotation
*Career choices amid constraints*	
1. Making health‐conscious career decisions	My heart rate isn't particularly fast, but it feels as though something is wrapped around my heart, and with each beat, there's a sensation of throbbing pain. Sometimes, my heart beats for a few times at a normal rate, followed by a single beat that brings that throbbing pain. In the mornings, I might feel this throbbing pain for two or three beats, but once I put on some clothes and warm up, the sensation goes away. (2–19) Because I have a stent, the doctor is concerned about the risk of blood clots and poor blood flow. However, since I take anticoagulants, my skin bruises easily. At first, I thought it was just from me lightly touching my skin, and it felt a bit unfamiliar. (7–11)
2. Adapting one's careers to one's personal interests	The reason I'm willing to stay and continue working is because some things in this job are things I enjoy, which helps me push through difficult times and keep going despite the challenges. (11–11)
*Adjustments to one's work environment for reasons of fatigue*	
1. Balancing work and health through self‐awareness	…. My job requires a lot of movement—delivering meals, carrying food and introducing the menu. Even though there's a one‐hour break in the middle, after four consecutive days of work, my body can't keep up and I ended up feeling unwell for a while. I realised I couldn't handle the physical demands of full‐time work. However, with hourly work, the total hours are similar to full‐time and the flexible schedule allows my body to manage it. (10–17)
2. Maintaining physical endurance for work	If my work schedule is regular, I rarely experience heart pain throughout the month. But if I suddenly stay up late or follow an irregular schedule for a week, my condition worsens during that period. Sometimes, I may even experience sudden heart pain in the middle of the night…. (2–34)
	… I can't let myself slack off on weekends… I need to go for a walk to maintain physical activity and prevent my body from deteriorating too quickly. I think exercise is really important. Even if it's not hiking, just brisk walking or walking around a track or a small park is beneficial. (15–22)
3. Choosing low‐pressure and flexible work environments	For my condition, there are many jobs I can do, but only a few allow me to work without too much pressure. (15–15)
	When I wake up in the morning, I often feel intense chest pain, along with noticeable tightness and shortness of breath. I usually need to take unscheduled leave to allow the symptoms to ease, and once I feel stable, I go to work. (8–26)
*Crises in the workplace arising from exceeding one's physical limits*	
1. Life‐threatening, irreversible heart failure from demands in the workplace	In the past, when my condition worsened, I would occasionally have trouble breathing and feel more easily winded. It felt like my blood circulation wasn't good, and I felt quite uncomfortable. (4–41)
2. Frequent sick leave due to worsening health	When my health is really bad, even stepping out of the house is difficult. Sometimes, just walking to the bathroom makes me start gasping, so I know I won't be able to go to work that day. (2–7)
3. Escalating symptoms requiring urgent medical intervention	A couple of times on my way to work, I experienced sharp chest pain that was very uncomfortable and I couldn't stand up, so I called an ambulance to take me to the hospital. (5–4)
4. Complications worsening the impact of workplace demands on heart health	I later developed arrhythmia, and every time I visited the doctor, they would say that arrhythmia cannot be cured and will stay with me for life. Over the past few years, I fainted twice, so I now have an internal defibrillator implanted. For me, it's a huge source of stress. I'm very anxious, not knowing when my heart rhythm will become irregular or when the defibrillator will shock me. It's an invisible kind of pressure. (18–16)
*Supportive networks for better health and job stability*	
1. Encouragement from family for better fitness and workplace adaptation	My mum also told me that, given my health condition, continuing with this job will only weaken me physically. My body isn't as young as it used to be, so she's worried that after working for so long, I might not be able to handle it. The doctor had told us that even though my health seems fine now, we don't know how long it will stay stable, or how long my heart can endure. Although I feel I should continue this job for a while, my mum is concerned and hopes I'll switch to a typical office job. She said those jobs are easier, with a 9‐to‐5 schedule, and at least I wouldn't have to run around. She wants me to do something less physically demanding. (10–20)
2. Support from supervisors through flexible work arrangements	Because of our work at the petrol station, I often have to run around helping with fueling, and sometimes my body feels uncomfortable. I inform my colleagues, and the boss's wife will suggest to the boss that he take over my shift so I can rest for a while. (5–6)
3. Assistance from colleagues through task sharing and emotional support	I believe that with congenital heart disease, working in any industry is manageable, as long as the workplace has colleagues who are willing to be understanding. For us, that is very important. The colleagues I have now are very willing to accommodate me. (15–13)
	The afternoon I was told I needed surgery, I received messages and articles from other colleagues about the high success rate of valve replacement surgeries. I was really touched because everyone was concerned about me and didn't see me as a burden. (4–37)
4. Support from the company for a more sustainable work arrangement	….once I returned home to rest, I handled tasks using my computer and negotiated with the company to adjust my rest period so I could work from home or take annual leave. The company was considerate and allowed me to work from home during my recovery. (7–39)
*Resilience at work for balance and fulfilment in life*	
1. Confidence and resilience in one's career	Maintain an optimistic attitude toward job hunting, set goals, and don't let negative emotions affect you. Think about the positive aspects work can bring, and even if you leave a job, it's an opportunity to move on to a more suitable workplace. (14–9)
	My biggest frustration right now is that, due to the pandemic, I have to wear a mask at work. The mask causes a slight lack of oxygen, so when my team leader or supervisor assigns me tasks, I don't always understand what they're saying after thinking it over. I have to ask them to repeat themselves before I can grasp what they mean. That's the part I find most frustrating. (15–21)
2. Thriving and finding meaning in one's career	I really enjoy early childhood education, so I'm happy to apply my expertise in the workplace. (4–6)
3. Balancing work, life, and future medical planning	I feel that illness has a significant impact on work, especially on livelihood. The reality is money. Since I was born with a congenital condition, I couldn't get insurance as a child, so I have to rely on money to cover medical expenses or emergency hospitalisation costs. If I don't have money, I won't be able to receive treatment. It's a very real situation. (8–31)
	I′ ve always believed that it's enough to have enough money, as long as it doesn't affect the quality of my life. (3–42)
4. Achieving fulfilment at work and contributing to the success of the team	From the client perspective, the sense of achievement comes when the elderly receive proper care through our services. From the employee perspective, it's when they feel happy, comfortable, and experience smooth work in this environment. These are all sources of my sense of accomplishment in my job. (9–49)

*Note:* Numbers in brackets at the end of each quote represent the participant number and the meaning unit number.

#### 5.2.1 Theme 1: Career Choices Amid Constraints

This theme illustrates how ACHD, despite facing physical limitations and the risk of disease progression, made career decisions that reflected both self‐awareness of their health status and sustained personal aspirations. It encompasses two subthemes: ‘making health‐conscious career decisions’—reflecting perseverance of effort in balancing health needs with work demands—and ‘adapting one's career to one's personal interests’—demonstrating consistency of interest as participants remained committed to long‐held passions despite setbacks. These choices highlight participants' capacity to pursue meaningful work aligned with both their physical realities and enduring motivations.

##### 5.2.1.1 Making Health‐Conscious Career Decisions

This subtheme cluster explores how ACHD select careers in consideration of their physical capacity while prioritising their health. This subtheme comprised three categories: ‘limitations in daily life due to breathlessness and pain’, ‘adapting one's lifestyle due to medication side effects, especially anticoagulants and bleeding risks’ and ‘choosing work within one's physical capacity’. Participants described experiencing fatigue, shortness of breath, chest discomfort and pain in the workplace. For instance, one participant reported experiencing morning chest tightness and breathing difficulties, especially during weather changes, sometimes accompanied by insomnia and arrhythmia *(2–19)*. In addition, those taking long‐term anticoagulants expressed concern regarding workplace injuries that could cause bleeding *(7–11)*. Selecting an appropriate job required them to carefully assess their physical capacity.

##### 5.2.1.2 Adapting One's Careers to One's Personal Interests

Many ACHD chose a job that aligned with their personal interests, and this gave them the intrinsic motivation to persist in the face of difficulties. One participant noted that a strong passion for their work helped them to continue even when encountering workplace difficulties *(11–11)*.

#### 5.2.2 Theme 2: Adjustments to One's Work Environment for Reasons of Fatigue

This theme highlights how ACHD adapted their work environments and routines in response to fatigue and physical limitations to sustain long‐term employment. The subthemes reflect both perseverance of effort and consistency of interest. ‘Balancing work and health through self‐awareness’ and ‘maintaining physical endurance for work’ show perseverance of effort, as participants continually adjusted strategies and exerted effort to remain productive despite physical challenges. ‘Preferring low‐pressure and flexible work’ environments illustrates consistency of interest, as participants maintained engagement in meaningful work by proactively choosing conditions that allowed them to pursue their goals sustainably.

##### 5.2.2.1 Balancing Work and Health Through Self‐Awareness

One participant, upon recognising that they could not tolerate intensive workloads, adjusted their work schedule by distributing their hours more evenly to ensure that they can perform at work without compromising their health *(10–17)*.

##### 5.2.2.2 Maintaining Physical Endurance for Work

This subtheme had three categories: ‘maintaining a consistent work schedule and having designated rest days’; ‘residing near one's workplace to reduce fatigue from commuting’; and ‘having an exercise and health maintenance routine’. A stable routine was considered essential for preventing physical discomfort. One participant reported experiencing chest tightness and pain after staying up late or following an irregular schedule *(2–34)*. To enhance physical endurance, participants engaged in physical activities such as regular exercise or traditional therapies. One individual, for instance, woke up at the same time every weekend to exercise *(15–22)*.

##### 5.2.2.3 Choosing Low‐Pressure and Flexible Work Environments

This subtheme illustrates how ACHD prioritise sustainable employment that corresponds to their capabilities while also allowing them to manage physical discomfort. It includes two categories: ‘selecting low‐pressure, stable roles’ and ‘seeking flexible work arrangements that allow for breaks or time off as needed’. One participant chose a low‐pressure job despite having many options *(15–15)*, whereas another struggled to arrive to work on time when the weather had wide fluctuations in temperature *(8–26)*.

#### 5.2.3 Theme 3: Crises in the Workplace Arising From Exceeding One's Physical Limits

This theme underscores the severe physical and mental toll on ACHD when workplace demands exceed their capabilities, potentially leading to life‐threatening health crises. Despite these challenges, participants' experiences reflect perseverance of effort and, to some extent, a challenge to consistency of interest. ‘Life‐threatening, irreversible heart failure from demands in the workplace’ and ‘frequent sick leave due to worsening health’ demonstrate perseverance of effort as participants continued to exert themselves despite escalating health issues. However, ‘escalating symptoms requiring urgent medical intervention and complications and multiple diseases intensifying the impact on heart health and work’ reflect the limitations of consistency of interest, as these crises disrupt participants' ability to maintain engagement with their work over time. This theme highlights the conflict between sustained effort and the eventual need for adjustment when physical resilience is overwhelmed.

##### 5.2.3.1 Life‐Threatening, Irreversible Heart Failure From Demands in the Workplace

This subtheme highlights how sustained work‐related fatigue or excessive stress may lead to irreversible heart failure in ACHD. One participant reported experiencing breathlessness and poor circulation after enduring prolonged work fatigue over an extended period *(4–41)*.

##### 5.2.3.2 Frequent Sick Leave Due to Worsening Health

After surgery, long‐term recovery may prevent work. One participant developed heart failure, experiencing breathlessness while walking, making work impossible *(2–7)*.

##### 5.2.3.3 Escalating Symptoms Requiring Urgent Medical Intervention

This subtheme highlights the sudden onset of severe and uncontrollable symptoms in ACHD experiencing heart failure, such as intense pain or difficulty breathing, which may result in syncope and require emergency care. One participant recounted experiencing acute chest pain while at work and being transported to the emergency department by ambulance *(5–4)*.

##### 5.2.3.4 Complications Worsening the Impact of Workplace Demands on Heart Health

This subtheme describes how heart failure may result in complications that require life‐sustaining interventions, thereby further impairing physical functioning and introducing additional work‐related challenges. One participant who experienced arrhythmia and episodes of fainting described the psychological and practical burden of requiring an implantable defibrillator for survival, which significantly affected both their personal life and work *(18–16)*.

#### 5.2.4 Theme 4: Supportive Networks for Better Health and Job Stability

This theme highlights the critical role that supportive networks—comprising family members, colleagues, supervisors and workplace policies—play in enabling ACHD individuals to maintain their health and secure stable employment. These networks contribute to both perseverance of effort and consistency of interest. ‘Encouragement from family for better fitness and workplace adaptation’ and ‘support from supervisors through flexible work arrangements’ reflect perseverance of effort, as such support helps participants sustain the effort required to remain employed despite health challenges. ‘Assistance from colleagues through task sharing and emotional support and support from the company for a more sustainable work arrangement’ demonstrate consistency of interest, as they ensure that participants can continue pursuing their professional goals in a sustainable manner, with fewer disruptions caused by health issues.

##### 5.2.4.1 Encouragement From Family for Better Fitness and Workplace Adaptation

A family's encouragement of physical activity during childhood results in the development of stamina required for work in adulthood. Family members also provide emotional support, share household responsibilities and encourage career choices that are less physically demanding to protect the patient's long‐term health. One participant shared that their mother advised against pursuing physically demanding jobs to prevent any further deterioration to their health *(10–20)*.

##### 5.2.4.2 Support From Supervisors Through Flexible Work Arrangements

This subtheme explores the role of empathetic supervisors in supporting ACHD. Supervisors can also provide assistance during medical emergencies. They recognise the need for medical leave during health setbacks and provide reassurance. One participant reported receiving both permission and practical support from their supervisor when feeling unwell at work *(5–6)*.

##### 5.2.4.3 Assistance From Colleagues Through Task Sharing and Emotional Support

Supportive colleagues cover for the patient's absences due to illness, minimising disruption in the workplace. One participant emphasised the importance of colleagues' understanding in their work life *(15–13)*. Another participant also shared that they were deeply moved by the emotional support received from colleagues while facing health problems *(4–37)*.

##### 5.2.4.4 Support From the Company for a More Sustainable Work Arrangement

This subtheme highlights the company's commitment to supporting employee health by offering regular health check‐ups, access to medical consultations and wellness programmes. When health problems require surgery, flexible work arrangements and appropriate compensation are provided. One participant who was hospitalised due to worsening heart function was offered flexible work options and medical leave *(7–39)*.

#### 5.2.5 Theme 5: Resilience at Work for Balance and Fulfilment in Life

This theme illustrates the challenges faced by ACHD in securing a job and how some demonstrate confidence and perseverance in the face of setbacks. Despite these challenges, they find value, fulfilment and financial stability in their careers, all while balancing work, life and preparing for future medical needs. This theme encompasses both perseverance of effort and consistency of interest. ‘Confidence and resilience in one's career’ and ‘thriving’ and finding meaning in one's career’ showcase perseverance of effort, as participants consistently push through setbacks and difficulties to continue their professional journeys. ‘Balancing work, life and future medical planning’ demonstrates consistency of interest, as participants maintain a long‐term focus on both their career and health, ensuring a stable and sustainable future. ‘Achieving fulfillment at work and contributing to the success of the team’ reflects the ongoing commitment to their roles, illustrating consistency of interest as they derive satisfaction from both personal achievements and team contributions.

##### 5.2.5.1 Confidence and Resilience in One's Career

Despite their health challenges, ACHD should pursue career opportunities without self‐imposed limitations and view setbacks as part of their journey. Optimism and perseverance can lead to fulfilling careers, even when they face health challenges or have unmet goals. One participant approached work with a positive attitude *(14–9)*. The COVID‐19 pandemic reshaped work patterns, resulting in increased workloads and the need for workplace adaptations. Employees were required to adapt to new routines, including health protocols and task redistribution. One participant shared that wearing a mask during the pandemic affected their oxygen intake, requiring adjustments to maintain work performance *(15–21)*. Despite these challenges, ACHD demonstrated the capacity to find fulfilment and build meaningful careers.

##### 5.2.5.2 Thriving and Finding Meaning in One's Career

This subtheme illustrates how ACHD progress in their careers by identifying their goals, applying their expertise and making meaningful contributions. Through their efforts, they also inspire others to find value in their own work and to live purposefully. One participant expressed a sense of happiness at being able to use their professional skills in the workplace *(4–6)*.

##### 5.2.5.3 Balancing Work, Life and Future Medical Planning

This subtheme highlights that securing employment provides ACHD financial stability and the ability to save for future medical expenses. With a steady income, they strive to balance work and life, meeting job expectations without compromising their well‐being. One participant emphasised the importance of income for sustaining ongoing medical treatment *(8–31)*. Given the challenges associated with congenital heart disease, maintaining financial security through work and saving for health care becomes especially crucial when health setbacks affect earning capacity. Once job stability is established, individuals with ACHD tend to prioritise work–life balance instead of sacrificing their personal well‐being. One participant viewed their income as a means to achieve a more balanced and fulfilling life *(3–42)*.

##### 5.2.5.4 Achieving Fulfilment at Work and Contributing to the Success of the Team

This subtheme demonstrates that ACHD find fulfilment and a sense of achievement at work by completing tasks, reaching goals, receiving recognition, earning rewards, progressing in their careers and supporting their colleagues. For example, one participant described experiencing a sense of achievement and fulfilment through helping others in their work *(9–49)*.

## 6 Discussion

This is the first qualitative study to explore the essence of grit in the life experiences of individuals with chronic illness, specifically ACHD within the workplace. It identifies five key themes related to the expression of grit among ACHD in the workplace and provides empirical insight into how grit contributes to both job and life satisfaction. The findings reveal that grit, as experienced by ACHD, is a dynamic and adaptive trait shaped by the continuous negotiation between perseverance and adaptability. Rather than merely enduring hardship, ACHD demonstrate grit through their sustained commitment to personal values and long‐term professional goals, even amid ongoing health‐related uncertainty. This synthesis positions grit as a form of existential resilience—enabling individuals to maintain occupational identity and engagement while managing the complexities of chronic illness. These insights have important implications for nursing practice, highlighting the need to support the holistic well‐being and career development of this growing population.

Previous qualitative studies on ACHD have explored the impact of the condition on patients' quality of life (Ladak et al. 2020), perceptions and behaviours regarding physical activity (McKillop et al. 2018), and barriers and facilitators to the work experiences of young adults with CHD (Sluman et al. 2014). However, as the survival rate of ACHD patients increases, adulthood brings challenges related to employment and work. In this study, most participants had a NYHA classification > 2, and grit was evident in their lived experiences of adapting to work and meeting its demands.

This study provides a qualitative perspective on the complications of atrial arrhythmias in ACHD (Kerley et al. 2025) and the symptoms of fatigue or lack of energy, difficulty falling asleep, restless or excessive sleep and loss of motivation or enjoyment in activities associated with depression, as noted by Liu, Chung, et al. (2024). These symptoms were linked to physical limitations described by participants in their daily lives, such as breathlessness and pain, lifestyle changes due to medication side effects, or work‐related crises resulting from exceeding their physical limits. In particular, as their health deteriorates, frequent sick leave can impact employment and increase the risk of losing control over their condition. This phenomenon also highlights how grit manifests in the daily lives of ACHD patients, as they continue to pursue work they are passionate about while selecting jobs that align with their physical capabilities.

As disease severity increases, available job opportunities diminish and the likelihood of engaging in part‐time or secondary employment rises (Geyer et al. 2009). Grit plays a crucial role in enhancing employability traits, including career agility, cultural adaptability and proactive career development. It also supports the development of autonomy, leadership skills and personal employability (Ismail et al. 2023). This study reveals that ACHD often select occupations aligned with their personality and interests, demonstrating perseverance in the face of workplace challenges. Their passion for their chosen career motivates them to pursue ongoing learning and skill development, enabling them to sustain long‐term employment despite health‐related constraints.

Sluman et al. (2014) identified that ACHD individuals encounter workplace challenges stemming from their medical conditions and the need for extended recovery time, which aligns with the findings of this study. Participants reported experiencing physical symptoms while working and emphasised the necessity of adequate recovery periods. To meet occupational demands, they highlighted the importance of having autonomy in determining when to take rest. Grit has been recognised as a source of personal strength linked to proactive self‐management beliefs (McKelvey et al. 2023). This study found that grit enables ACHD individuals to recognise their limits, adapt accordingly and identify the importance of a work environment that minimises physical fatigue. It confirms that grit has a protective function, even in the presence of complications (Bonanni et al. 2025), frailty, and the risk of cognitive decline (Daelman et al. 2024). Therefore, when Cifra et al. (2025) discuss improving the quality of ACHD care through the integration of cardiopulmonary exercise within CHD lifecycle‐centred care, the concept of grit should be incorporated.

Heart failure further heightens the risk of recurrent arrhythmias (Lauwers et al. 2025); findings from the present study indicate that ACHD are acutely aware of the onset of heart failure symptoms in their daily work lives. They demonstrate grit in managing deteriorating health, adjusting to evolving workplace demands and adapting to new physical limitations. This study found that ACHD individuals demonstrate grit by taking responsibility for their health, facing physical deterioration with optimism, identifying the causes of illness early and seeking timely treatment. This aligns with the findings of Liu, Lu, et al. (2024), where higher scores were observed in the health‐promoting lifestyle aspects of interpersonal support, self‐actualisation and stress management. These insights reinforce the importance of equipping ACHD with tailored interventions and self‐management strategies (Acuña Mora et al. 2022) that support continued employment and enhance overall well‐being.

In Taiwan, research has indicated that chronic cardiovascular conditions negatively impact employment (Lin 2015), but this study further reveals that the expression of grit among ACHD in Taiwan is deeply rooted in sociocultural expectations that value perseverance, responsibility and achievement. Many participants described feeling compelled to maintain their careers despite physical limitations, driven by a sense of duty to support their families and fulfil societal standards of success. This cultural backdrop can enhance perseverance, as individuals strive to overcome health‐related obstacles to meet these obligations. However, when health deteriorates, such expectations may lead to role strain, particularly in work environments where expressing vulnerability or requesting accommodations is culturally discouraged. This aligns with Yang (2013) findings, which indicate that physical health status can affect work attendance and job position. CHD patients often face significant challenges in maintaining their health. This study found that early family environments have a lasting impact on health‐promoting behaviours in ACHD adulthood, with active family encouragement serving as a key supportive factor—aligning with previous research (Bay et al. 2021). These findings highlight the importance of workplace and family support in fostering grit and sustaining employment among ACHD individuals. Family members often serve as critical sources of emotional and practical support, helping to manage both healthcare needs and work‐related adjustments. These cultural norms may strengthen consistency of interest, as ACHD remain motivated to sustain employment for the stability and well‐being of their families. Ultimately, the Taiwanese cultural context fosters resilience strategies aligned with social expectations—such as silent endurance and prioritising work performance over personal care—revealing how grit in ACHD is shaped by the interplay of cultural values, family roles and chronic illness.

Organisational culture and the workplace environment play a critical role in job retention for ACHD individuals. In terms of fulfilling work demands, they value workplace interpersonal interactions, including a supportive work environment and good relationships with colleagues (Liu, Chung, et al. 2024). This study found that emotional support in adulthood, understanding supervisors who care for their condition, as well as colleagues who share tasks and provide emotional support, further strengthen the grit of ACHD individuals. Therefore, fostering an understanding and inclusive company culture can enhance job retention for ACHD individuals. At the organisational level, occupational physicians and company representatives should take a proactive approach in implementing and maintaining preventive measures for workplace health management (Bosma et al. 2021). In Taiwan, the Health Promotion Administration has shifted its focus toward workplace health promotion since 2003, establishing support centres and certifying over 12,000 workplaces by 2014. This shift also prompted the Ministry of Labour to revise the Occupational Safety and Health Act in 2013 to clarify employers' responsibilities in protecting and promoting workers' health (Chen and Yu 2016). However, the findings from this study highlight the need for holistic support systems that integrate welfare policies, labour regulations and workplace accommodations for individuals with chronic conditions such as ACHD. Enhancing workplace flexibility, targeted welfare support and inclusive labour policies can help individuals with chronic conditions balance work and health—reflecting Chang et al. (2021) view that workplace health culture and health promotion are closely linked, particularly through health policies, a supportive climate and peer and supervisor support. This approach not only supports their perseverance of effort in overcoming obstacles but also fosters consistency of interest in pursuing long‐term career and personal goals. Tailored support systems are essential to ensuring that individuals with chronic health conditions can continue contributing meaningfully to society while safeguarding their well‐being.

### 6.1 Strengths and Limitations

This qualitative study recruited participants from paediatric and ACHD outpatient clinics at two medical centres in northern Taiwan, with selection bias due to participant availability for interviews. The sample included individuals with diverse disease conditions and occupations, reflecting CHD heterogeneity. Both male and female participants were included, with age differences potentially influencing findings. The study focused solely on the ACHD perspective and did not include viewpoints from employers, family members, or healthcare providers. Data collection occurred during the COVID‐19 pandemic, leading to increased use of virtual interviews. As a qualitative study, the findings do not directly address the higher unemployment rate or provide specific solutions to work‐related challenges for ACHD individuals.

### 6.2 Recommendations for Further Research

Future research should investigate how grit, as an element of positive psychological capital, can be effectively applied in the management of chronic diseases. As individuals with CHD age, studies should extend beyond adulthood to include older populations, with particular attention to promoting successful ageing. Further research is also needed to examine the role of grit‐based interventions in CHD care, including areas such as the impact of pregnancy on employment, recovery following cardiac deterioration, genetic counselling, insurance coverage and retirement planning.

### 6.3 Implications for Policy and Practice

This study's findings suggest that incorporating the concept of grit into care plans for individuals transitioning from adolescence to adulthood may enhance support for ACHD. Given the range of challenges ACHD encounter in adulthood, integrating grit into transitional care planning could offer meaningful benefits. Furthermore, embedding grit‐focused strategies within employment readiness programmes may provide more comprehensive support for ACHD as they enter the workforce. These insights underscore the importance of developing tailored interventions that foster perseverance, with the aim of improving both career stability and overall well‐being. The findings also highlight the need for policy and practice frameworks that address the psychological and practical dimensions of support for ACHD.

## 7 Conclusion

Grit plays a vital role in enhancing life satisfaction and work performance among ACHD, significantly shaping their experiences in the workplace. These individuals demonstrate perseverance in securing employment, adjusting work strategies to manage physical fatigue, overcoming health‐related crises and pursuing personal fulfilment, while relying heavily on family and social support. The findings underscore the importance of perseverance and mental well‐being in both professional and everyday life for ACHD, offering valuable insights to inform psychological support and targeted interventions. Coping strategies, such as modifying the work environment and managing fatigue, assist in alleviating workplace stress. In addition, interpersonal support, particularly from family and social networks, proves to be positively influential in job retention.

## Author Contributions

All authors made substantial contributions. Yu‐Shiu Liu, Jou‐Kou Wang and Chi‐Wen Chen were responsible for the study conception and design. All authors participated in the data collection and analysis. Yu‐Shiu Liu and Chi‐Wen Chen wrote the article with the input from all authors. Chun‐Wei Lu, Hung‐Tao Chung, Jou‐Kou Wang, Ying‐Mei Hsu and Chi‐Wen Chen supervised the study. All authors approved the final version for submission.

## Conflicts of Interest

The authors declare no conflicts of interest.

## Supporting information


**Appendix S1.** Consolidated Criteria for Reporting Qualitative Studies (COREQ) reporting guideline.

## Data Availability

Data available on request due to ethical restrictions.
