# Supplementary material for: Grit in the Workplace Experienced by Taiwanese Adults With Congenital Heart Disease: A Phenomenological Study
Source: J Clin Nurs. 2025 Jul 22;35(2):866–78. doi: 10.1111/jocn.70051 (PMC12779234; doi:10.1111/jocn.70051)
Supplement: Supplementary file 1 — Appendix S1. Consolidated Criteria for Reporting Qualitative Studies (COREQ) reporting guideline. [file JOCN-35-866-s001.docx]

Appendix S1 Consolidated Criteria for Reporting Qualitative Studies (COREQ) reporting guideline

| No Item | Guide questions/description | Page |
| --- | --- | --- |
| Domain 1: Research team and reflexivity |  |  |
| Personal Characteristics |  |  |
| 1. Interviewer/facilitator | Which author/s conducted the interview? | Page 10-11 |
| 1. Credentials | What were the researcher’s credentials? E.g. PhD, MD | Page 14 |
| 1. Occupation | What was their occupation at the time of the study? | Page 14 |
| 1. Gender | Was the researcher male or female? | non |
| 1. Experience and training | What experience or training did the researcher have? | Page 15-16 |
| Relationship with participants |  |  |
| 1. Relationship established | Was a relationship established prior to study commencement? | Page 12 |
| 1. Participant knowledge of the interviewer | What did the participants know about the researcher? e.g. personal goals, reasons for doing the research | Page 12-13 |
| 1. Interviewer characteristics | What characteristics were reported about the interviewer/facilitator? e.g. Bias, assumptions, reasons and interests in the research topic | Page 12-13 |
| Domain 2: study design |  |  |
| Theoretical framework |  |  |
| 1. Methodological orientation and Theory | What methodological orientation was stated to underpin the study? e.g. grounded theory, discourse analysis, ethnography, phenomenology, content analysis | Page 9-10 |
| Participant selection |  |  |
| 1. Sampling | How were participants selected? e.g. purposive, convenience, consecutive, snowball | Page 10-11 |
| 1. Method of approach | How were participants approached? e.g. face-to-face, telephone, mail, email | Page 10-11 |
| 1. Sample size | How many participants were in the study? | Page 12-13 |
| 1. Non-participation | How many people refused to participate or dropped out? Reasons? | non |
| Setting |  |  |
| 1. Setting of data collection | Where was the data collected? e.g. home, clinic, workplace | Page 12-13 |
| 1. Presence of non-participants | Was anyone else present besides the participants and researchers? | Page 12-13 |
| 1. Description of sample | What are the important characteristics of the sample? e.g. demographic data, date | Page 12-13 |
| Data collection |  |  |
| 1. Interview guide | Were questions, prompts, guides provided by the authors? Was it pilot tested? | Page 12 |
| 1. Repeat interviews | Were repeat interviews carried out? If yes, how many? | non |
| 1. Audio/visual recording | Did the research use audio or visual recording to collect the data? | Page 12-13 |
| 1. Field notes | Were field notes made during and/or after the interview or focus group? | Page 12-13 |
| 1. Duration | What was the duration of the interviews or focus group? | Page 12-13 |
| 1. Data saturation | Was data saturation discussed? | Page 12-13 |
| 1. Transcripts returned | Were transcripts returned to participants for comment and/or correction? | Page 14-15 |
| Domain 3: analysis and findings |  |  |
| Data analysis |  |  |
| 1. Number of data coders | How many data coders coded the data? | Page 16-17 |
| 1. Description of the coding tree | Did authors provide a description of the coding tree? | Page 17-26 |
| 1. Derivation of themes | Were themes identified in advance or derived from the data? | Page 17-26 |
| 1. Software | What software, if applicable, was used to manage the data? | non |
| 1. Participant checking | Did participants provide feedback on the findings? | Page 14-15 |
| Reporting |  |  |
| 1. Quotations presented | Were participant quotations presented to illustrate the themes / findings? Was each quotation identified? e.g. participant number | Page 17-26 |
| 1. Data and findings consistent | Was there consistency between the data presented and the findings? | Page 17-26 |
| 1. Clarity of major themes | Were major themes clearly presented in the findings? | Page 17-26 |
| 1. Clarity of minor themes | Is there a description of diverse cases or discussion of minor themes? | Page 17-26 |
